# Postbiotics engage IRF4 in adipocytes to promote sex‐dependent changes in blood glucose during obesity

**DOI:** 10.14814/phy2.15439

**Published:** 2022-08-22

**Authors:** Brittany M. Duggan, Anita M. Singh, Darryl Y. Chan, Jonathan D. Schertzer

**Affiliations:** ^1^ Department of Biochemistry and Biomedical Sciences McMaster University Hamilton Canada; ^2^ Farncombe Family Digestive Health Research Institute McMaster University Hamilton Canada; ^3^ Centre for Metabolism, Obesity and Diabetes Research McMaster University Hamilton Canada

**Keywords:** adipocyte, glucose, immunometabolism, insulin, IRF4, postbiotics

## Abstract

Postbiotics are microbial‐derived components or metabolites that can influence host immunity and metabolism. Some postbiotics can improve blood glucose control and lower inflammation during bacterial or nutritional stress. Bacterial cell wall‐derived muramyl dipeptide (MDP) is a potent insulin‐sensitizing postbiotic that engages NOD2, RIPK2, and requires interferon regulatory factor 4 (IRF4) to lower inflammation and improve blood glucose. However, the sex‐dependent effects of this postbiotic and the cell type required for IRF4 to cause inflammatory versus glycemic responses to MDP were unknown. Here, we measured how MDP injection altered glucose tolerance and adipose tissue inflammation during low‐level endotoxemia and high fat diet (HFD)‐induced obesity in male and female adipocyte‐specific IRF4 knockout mice (AdipoIRF4^fl/fl^) compared to WT^fl/fl^ mice. Adipocyte IRF4 was required for the blood glucose‐lowering effects of MDP during endotoxemia and HFD‐induced obesity in male mice. However, MDP did not alter blood glucose in female WT^fl/fl^ and AdipoIRF4^fl/f^ mice during endotoxemia. Unexpectedly, female HFD‐fed AdipoIRF4^fl/f^ mice had lower blood glucose after MDP treatment compared to WT^fl/fl^ mice. MDP lowered inflammatory gene expression in adipose tissue of HFD‐fed WT^fl/fl^ and AdipoIRF4^fl/fl^ mice of both sexes. Therefore, MDP‐mediated lowering of adipose inflammation does not require adipocyte IRF4 and was independent of sex. Together, these data show that injection of MDP, an insulin‐sensitizing postbiotic, lowers adipose tissue inflammation in male and female mice, but lower adipose inflammation is not always associated with improved blood glucose. The blood glucose‐lowering effect of the postbiotic MDP and dependence on adipocyte IRF4 is sex‐dependent.

## Graphical abstract

Postbiotics from the gut microbiota can alter host metabolism. It was not clear how certain postbiotics improved blood glucose control. Here, it was found that a bacterial cell wall postbiotic improved blood glucose via interferon regulatory factor 4 (IRF4) transcription factor within adipocytes. IRF4 did not alter inflammation responses in adipose tissue, but rather worked in a sex‐dependent manner to alter blood glucose in response to postbiotics.

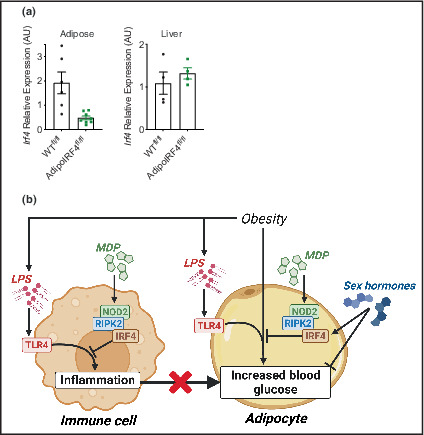

## 1 INTRODUCTION

Postbiotics include microbial components or metabolites that do not require detection of live bacteria to influence host immunity and metabolism (Cavallari et al., 2017; Cavallari et al., 2020; Cicenia et al., 2014; Patel & Denning, 2013). For example, microbial components sensed by pattern recognition receptors (PRRs) of the innate immune system can alter host metabolism (Canfora et al., 2015; Cani et al., 2007; Duggan et al., 2022; Perry et al., 2016; Tran et al., 2019; Turnbaugh et al., 2006). Specific postbiotics, such as bacterial cell wall‐derived muramyl dipeptide (MDP), have been shown to mitigate metabolic inflammation and improve aspects of metabolic disease during bacterial and nutritional stress (Canfora et al., 2015; Cavallari et al., 2017; Cavallari et al., 2020; Tran et al., 2019). Previous work has defined MDP as a potent insulin‐sensitizing postbiotic that engages Nucleotide‐binding oligomerization domain‐containing protein 2 (NOD2)‐Receptor interacting serine/threonine protein kinase 2 (RIPK2) and is regulated by interferon regulatory factor 4 (IRF4) (Cavallari et al., 2017; Cavallari et al., 2020). NOD2 activation after injection of MDP promotes immune tolerance, lowers adipose tissue inflammation, and improves blood glucose control during low‐dose endotoxin challenge and during obesity (Cavallari et al., 2017). We have shown that these inflammation‐ and glucose‐lowering effects were dependent on non‐hematopoietic RIPK2, which was absent in male mice with a whole‐body deletion of IRF4. However, the cell type responsible for IRF4‐mediated effects of the insulin‐sensitizing postbiotic, MDP and the sex‐dependent effects on blood glucose homeostasis and adipose inflammation were not known. Here, we tested if adipocyte IRF4 was required for MDP‐induced changes in blood glucose control and adipose inflammation in male and female mice during low‐level endotoxin stress or diet‐induced obesity. Our results show that adipocyte IRF4 is required for the postbiotic MDP to improve blood glucose during endotoxemia or obesity in male mice. However, female mice were refractory to glycemic effects of MDP. We uncovered a sex‐dependent effect of IRF4 deletion on blood glucose control during postbiotic treatment in obese mice. MDP lowered adipose tissue inflammation in male and female obese mice. Therefore, lower inflammation is not required for the sex‐dependent effects of the postbiotic MDP on blood glucose.

## 2 METHODS

### 2.1 Mice

All animal procedures were approved by the Animal Research Ethics Board of McMaster University and all mice were housed in 12 h light‐fark conditions with ad libitum access to water and food (standard CD, cat# 8640; Teklad 22/5 or HFD, cat# D12492, Research Diets, 60% kcal from fat, as specified). Adipocyte‐specific *Irf4* ^*−/−*^ animals were generated by crossing adiponectin‐cre (adipoq‐cre) transgenic mice (cat# 028020; The Jackson Laboratory) with IRF4^loxP/loxP^ mice (cat# 009380; The Jackson Laboratory). IRF4^loxP/loxP^ mice lacking the adipoq‐cre transgene were controls for all experiments. Adipocyte *Irf4* deletion was confirmed by tail DNA genotyping. Mouse tail clipping (2‐3 mm) were digested using Wisent Advanced DNA Fast Extract DNA kit (cat# 801‐200‐HR) according to manufacturer's instructions. The presence of the adipoq‐cre transgene was confirmed by polymerase chain reaction (PCR) amplification of isolated DNA. Primers for the adipoq‐cre transgene (3′ primer ACG GAC AGA AGC ATT TTC CA and 5′ primer GCA TGT GCC ATH THA GTC TG; amplifies only when adipoq‐cre is present) and a PCR internal control amplification band (3′ primer GTA GGT GGA AAT TCT AGC ATC ATC C and a 5′ primer CTA GGC CAC AGA ATT GAA AGA TCT; amplifies for all reactions) were used in a single reaction. In a separate reaction, the presence of IRF4^loxP/loxP^ was also confirmed by PCR amplification of isolated DNA (WT 3′ primer CTC TGG GGA CAT CAG TCC T; Floxed 3′ primer CGA CCT GCA GCC AAT AAG C; Common primer TGG GCA CCT CTA CTG TCT GG, selected according to Jackson Laboratory genotyping protocol).

### 2.2 Postbiotic treatment during low‐level endotoxemia and obesity

For acute endotoxemia experiments, all mice were fed a standard chow diet (cat# 8640 Teklad 22/5; Envigo) for duration of experiments. At 8–12 weeks of age, mice were injected with MDP (100 μg, i.p.; Invivogen, cat# tlrl‐mdp) for 3 days. On the 4th day, 0.2 mg/kg Ultrapure LPS derived from *E. coli* (Invivogen, Cat #tlrl‐3pelps) was injected (i.p) 6 h prior to a GTT. For dietary‐induced obesity studies, mice were switched from standard chow to a diet containing 60% kcal fat (cat# D12429; Research Diets) at 9–12 weeks of age, which coincided with the onset of chronic MDP injections. Mice were fed ad libitum for 5 weeks and MDP (100 μg, i.p) was injected 4 days/week during the 5‐week period. At the end of the 4th week, a GTT was performed and at the end of the 5th week, epidydimal white adipose tissue (WAT) was collected, immediately flash frozen in liquid nitrogen and stored at −80°C. All GTTs were performed using i.p. injections in 6 h fasted mice and glucose was determined by tail vein blood sampling using a handheld glucometer (Roche). Detailed procedures have been published (Anhê et al., 2022).

### 2.3 Adipose tissue and liver inflammatory gene expression

Total RNA was obtained from ∼50 mg of epidydimal WAT or liver via mechanical homogenization at 4.5 m/s for 45 s using a FastPrep‐24 tissue homogenizer (MP Biomedicals) and glass beads, followed by phenol‐chloroform extraction. RNA was treated with DNase I (Thermo Fisher Scientific) and cDNA was prepared using 500–1000 ng total RNA and SuperScript III Reverse Transcriptase (Thermo Fisher Scientific). Transcript expression was measured using TaqMan Assays with AmpliTaq Gold DNA polymerase (Thermo Fisher Scientific) and target genes were compared to the mean of *Rplp0* and *18S* housekeeping genes using the ΔΔ*C*_T_ method.

### 2.4 Statistical analysis

Values are presented as mean ± SEM. Data were assessed for normal distribution using the D'Agostino‐Pearson normality test. Statistical significant (*p* < 0.05) was determined by unpaired two‐tailed t‐test for normally distributed data sets and by Mann–Whitney U‐test for non‐normally distributed data sets. Statistical analysis were performed with GraphPad Prism 8–9 software. Diagrams were created using BioRender.com software.

## 3 RESULTS

### 3.1 Adipocyte IRF4 dictates a sex‐specific blood glucose response to MDP during low‐level endotoxemia

**FIGURE 1:**
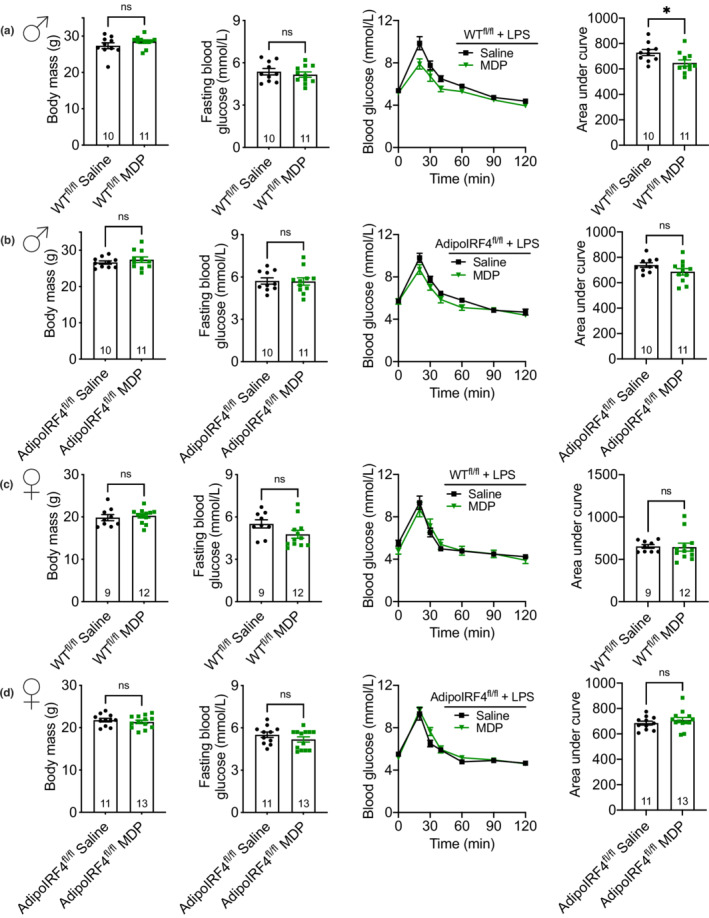
Adipocyte IRF4 dictates a sex‐specific glucose response to MDP during endotoxemia. Body mass, FBG, blood‐glucose vs. time and area under the blood glucose curve during a GTT (2 g/kg, i.p.) in (a) male WT^fl/fl^, (b) male AdipoIRF4^fl/fl^, (c) female WT^fl/fl^ and (d) female AdipoIRF4^fl/fl^ mice injected with saline or MDP (100 μg, i.p) for 3 days and before low‐dose LPS injection (0.2 mg/kg, i.p.) on day 4, 6 h before glucose tolerance test was performed (*n* = 9‐13/group). Each dot is a separate mouse.

We have previously shown that administration of MDP can lower blood glucose after a glucose load (i.e., glucose tolerance test) during an acute, low‐level endotoxin challenge, and that mice with whole‐body deletion of RIPK2, NOD2 or IRF4 are refractory to the effects of MDP on blood glucose (Cavallari et al., 2017; Cavallari et al., 2020). Here we tested the blood glucose‐lowering effect of MDP in male and female mice with an adipocyte‐specific *Irf4* ^*−/−*^ deletion (AdipoIRF4^fl/fl^) compared to mice lacking the adiponectin‐cre transgene (WT^fl/fl^). Male and female WT^fl/fl^ and AdipoIRF4^fl/fl^ mice were injected with MDP for 3 days prior to low dose LPS injection followed by 6 h fasting and a glucose tolerance test (GTT). We found that administration of MDP lowered blood glucose levels during the GTT in WT^fl/fl^, but not AdipoIRF4^fl/fl^ male mice (Figure 1a,b), demonstrating a requirement for adipocyte IRF4 in propagating the glucose‐lowering effects of MDP injection during low‐level endotoxin stress. However, we did not observe lower blood glucose levels in female WT^fl/fl^ or AdipoIRF4^fl/fl^ after MDP administration (Figure 1c,d), showing a sex‐specific effect of this postbiotic on glucose metabolism during low‐level endotoxemia.

### 3.2 Adipocyte IRF4 dictates a sex‐specific blood glucose response to MDP during diet‐induced obesity

**FIGURE 2:**
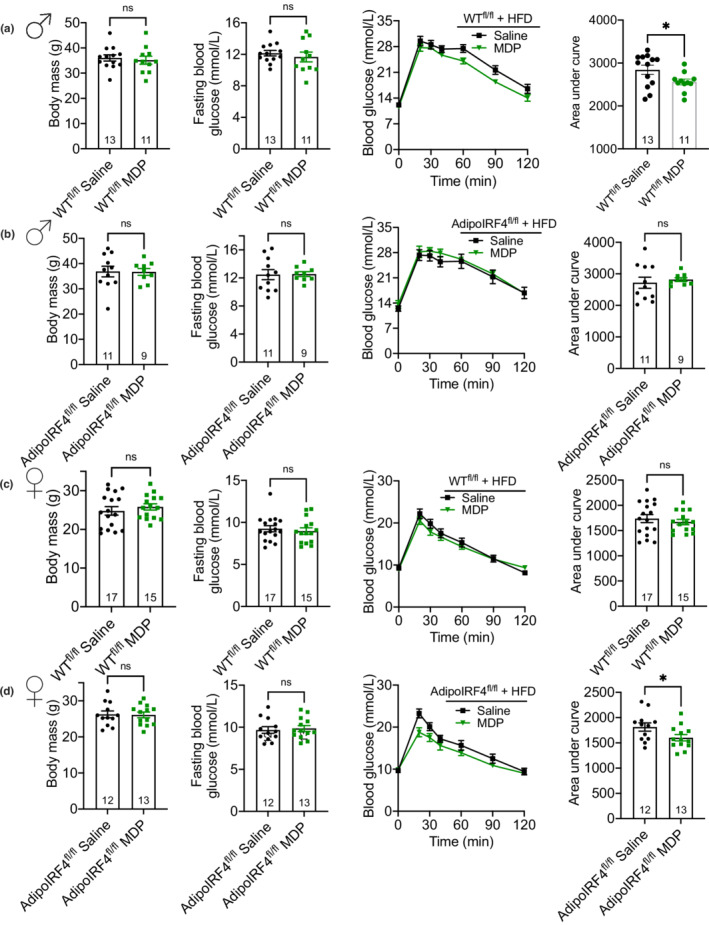
Adipocyte IRF4 dictates a sex‐specific glucose response to MDP during obesity. Body mass, FBG, blood‐glucose vs. time and area under the blood glucose curve during a GTT (1.5 g/kg, i.p.) in (a) male WT^fl/fl^, (b) male AdipoIRF4^fl/fl^, (c) female WT^fl/fl^ and (d) female AdipoIRF4^fl/fl^ mice fed a 60% HFD and injected with saline or MDP (100 μg, i.p, 4d/wk) for 4 weeks (*n* = 11‐17/group). Each dot is a separate mouse.

We have previously shown that chronic NOD2 activation with MDP lowers blood glucose during a glucose tolerance test, when the postbiotic MDP is injected 4 days per week for 5 weeks, co‐initiated with the onset of HFD‐feeding (Cavallari et al., 2017). Consistent with our previously published results MDP did not alter blood glucose in obese male mice with a whole‐body deletion in IRF4 treated with MDP (Cavallari et al., 2017). Therefore, we next tested if adipocyte IRF4 was required for MDP to improve blood glucose control during HFD‐feeding. Consistent with our previously published results (Cavallari et al., 2017; Cavallari et al., 2020), we found that HFD‐fed, male WT^fl/fl^ mice treated with MDP (4 days per week) had significantly lower blood glucose levels during a GTT, evinced by a significantly lower area under the blood glucose‐time curve (Figure 2a). In contrast, there was no difference in blood glucose levels in HFD‐fed, male AdipoIRF4^fl/fl^ mice treated with MDP (Figure 2b), supporting a role for adipocyte IRF4 in mediating the glucose‐lowering effects of MDP during obesity in male mice. Female WT^fl/fl^ mice treated with MDP showed no difference in glucose levels during a GTT (Figure 2c). Interestingly, we found that MDP lowered blood glucose in female AdipoIRF4^fl/fl^ mice, which had significantly lower area under the blood glucose‐time curve (Figure 2d). Taken together, our data support a role for adipocyte IRF4 in mediating the glucose‐lowering effects of the postbiotic MDP during diet‐induced obesity in male mice. However, the glucose‐lowering effects of MDP injection and adipocyte IRF4 deletion are sex‐dependent in obese mice.

### 3.3 MDP lowers adipose inflammation independent of sex and adipocyte IRF4

**FIGURE 3:**
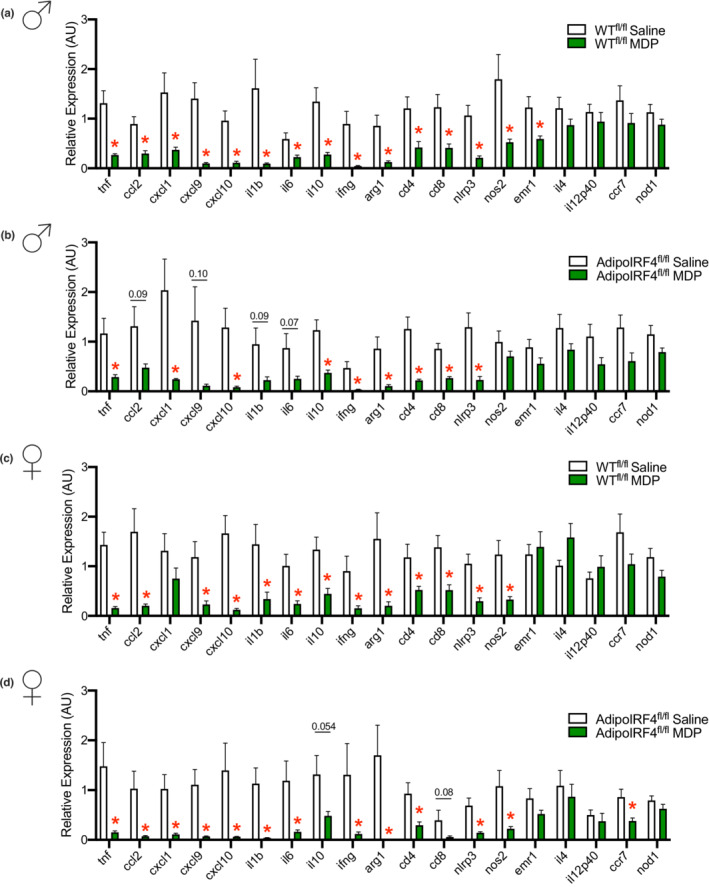
MDP lowers markers of adipose inflammation in a sex‐independent and adipocyte IRF4‐independent manner. Transcript levels of inflammatory and immune markers in white adipose tissue of (a) male WT^fl/fl^, (b) male AdipoIRF4^fl/fl^, (c) female WT^fl/fl^ and (d) female AdipoIRF4^fl/fl^ mice fed a 60% HFD and treated with saline or MDP (100 μg, i.p, 4d/wk) for 5 weeks (*n* = 8‐17/group).

**FIGURE 4:**
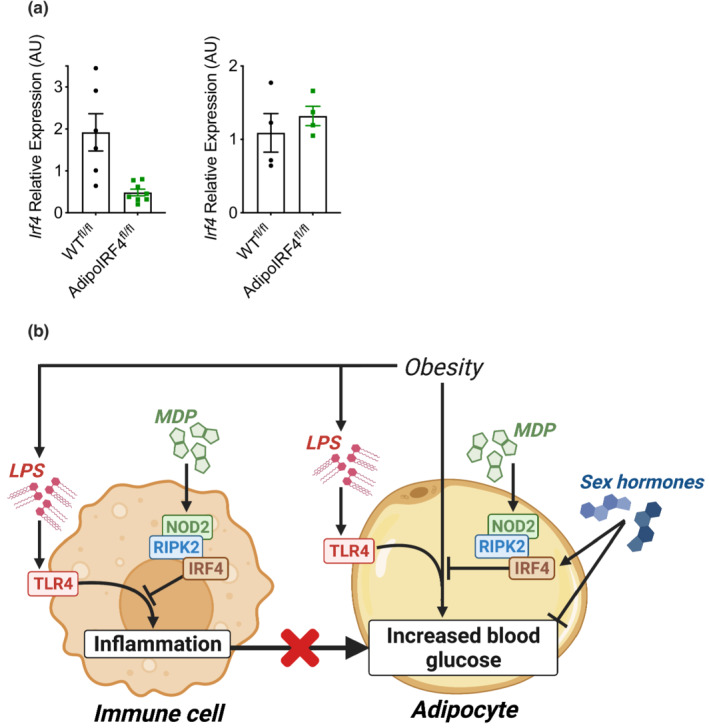
Role of adipocyte IRF4 in metabolic inflammation and blood glucose control. (a) AdipoIRF4^fl/fl^ model validation by quantification of transcript levels of *Irf4* in whole white adipose tissue (left) and liver (right) of WT^fl/fl^ and AdipoIRF4^fl/fl^ mice. (b) Immune cell IRF4 is positioned as a sex‐independent mediator of the anti‐inflammatory actions of MDP during low‐dose endotoxin and HFD‐induced obesity. However, lower adipose tissue inflammation is not always sufficient to promote improved blood glucose control. Adipocyte IRF4 mediates the glucose‐lowering effects of MDP during low‐dose endotoxin and HFD‐induced obesity in a sex‐dependent manner, but it is unknown how sex‐dependent factors influence adipocyte IRF4 and the glucose response to MDP.

We have previously shown that repeated administration of MDP lowers expression of inflammatory markers in adipose tissue of obese male mice, but adipose inflammation was not altered by MDP in obese male mice with a whole‐body deletion of IRF4 (Cavallari et al., 2017). It was not known if IRF4 in immune cells or adipocytes propagated the immune tolerizing effects of MDP. Here, we tested if adipocyte IRF4 was required for lowering of adipose inflammation after chronic injection of MDP in HFD‐induced obese male and female mice. Consistent with previous findings, we found that male WT^fl/fl^ had reduced expression of inflammatory genes in epidydimal adipose tissue after chronic MDP treatment (Figure 3a). Interestingly, a similar pattern of lower expression of many inflammatory markers occurred in both male WT^fl/fl^ and male AdipoIRF4^fl/fl^ mice after MDP treatment (Figure 3b), despite male AdipoIRF4^fl/fl^ mice being refractory to NOD2‐mediated glucose changes during low‐level endotoxemia (Figure 1b) and during obesity (Figure 2b). Female WT^fl/fl^ and AdipoIRF4^fl/fl^ mice also showed a similar pattern of lower inflammatory gene expression (Figure 3c,d). MDP consistently lowered many inflammatory markers in male and female mice of both genotypes, such as *tnf, ccl2, cxcl10, il1b, il6, il10, ifng, cd4, cd8, nlrp3*, and *nos2*. These results show that MDP‐mediated changes in inflammatory markers in adipose tissue do not parallel MDP‐mediated changes in blood glucose control, suggesting that lowered adipose tissue inflammation is not sufficient to mediate the beneficial effects of MDP on whole body glucose control. These results show that adipocyte IRF4 is dispensable for the anti‐inflammatory effect of MDP in adipose tissue during obesity. Consistent with these data, a previous report shows myeloid cell‐specific deletion of *Irf4* results in significantly increased expression of inflammatory markers in adipose tissue (Eguchi et al., 2013), highlighting a paracrine role for immune cell IRF4 regulating adipose tissue inflammation. We show that AdipoIRF4^fl/fl^ mice have ~75% lower IRF4 transcript levels in gonadal adipose tissue compared to WT^fl/fl^, but no change in the IRF4 transcript levels in the liver (Figure 4a). The totality of these results support the model proposed in Figure 4b.

## 4 DISCUSSION

Postbiotics are one class of microbial‐based interventions that can influence host metabolism and immunity. Postbiotics may circumvent some limitations of other microbial‐based approaches. Prebiotics, probiotics, antibiotics and fecal transfer may be limited by specificity, durability, and ability of microbes to find a niche in the gut for a sufficient duration to alter host physiology (Chen et al., 2021; Kristensen et al., 2016; Reijnders et al., 2016). Different postbiotics have contrasting effects on inflammation, insulin resistance and blood glucose homeostasis. For example, certain types of LPS and NOD1 ligands can promote insulin resistance and are shown to be augmented in circulation following high‐fat feeding or during obesity (Cani et al., 2007; Chan et al., 2017; Schertzer et al., 2011). In contrast, other postbiotics such as under‐acylated LPS, NOD2 ligands, short chain fatty acids (SCFA), flavonoids, and flagellin have emerged as promotors of insulin sensitivity and glucose homeostasis (Anhê et al., 2021; Canfora et al., 2015; Cavallari et al., 2017; Cavallari et al., 2020; Kimura et al., 2013; Thaiss et al., 2016; Tran et al., 2019). Recent work showed that injection of crude extracts of intestinal luminal contents, containing a complex mixture of postbiotics, improves blood glucose control in HFD‐fed mice (Duggan et al., 2022; Pomié et al., 2016). The identity and interactions of the microbial factors responsible for glucose lowering remain unknown. These studies demonstrate the need to understand how different postbiotics engage immune responses and alter host metabolism and how metabolic disease factors alter the host‐microbe relationship (Anhê et al., 2020).

Previous work has defined MDP as a potent insulin‐sensitizing postbiotic that engages NOD2, RIPK2, and IRF4 to alter metabolic inflammation and blood glucose control. Non‐hematopoietic RIPK2 mediates the glucose lowering and anti‐inflammatory effects of NOD2‐activating postbiotics (Cavallari et al., 2020). However, the specific cell type that mediated IRF4‐dependent effects on inflammation or blood glucose was not known. Here, we found that adipocyte IRF4 was required for the postbiotic MDP to convey improvements in blood glucose control in multiple models of bacterial or nutritional stress. One surprising finding was that female AdipoIRF4^fl/f^ mice had lower glucose after MDP treatment during obesity. Differences in male and female sex hormones are positioned to interact with the effect of MDP on IRF4 immunometabolism. IRF4 has been identified as an estrogen‐regulated gene in immune cells (Carreras et al., 2010) and estrogen receptor signaling has been shown to augment oxidative metabolism in adipose tissue (Zhou et al., 2020). Estrogen‐receptor signaling can increase IRF4 expression and drive distinct immune cell differentiation programs (Carreras et al., 2010), but it is not known how estrogen alters IRF4 expression or regulation of IRF4 transcriptional program in adipocytes. It is possible that estrogen levels and MDP interact through paracrine actions in adipocytes and adipose tissue resident immune cells to alter blood glucose control, which was revealed in WT^fl/fl^ versus AdipoIRF4^fl/fl^ female mice.

Another interesting result was that MDP lowered inflammatory gene expression in adipose tissue of obese WT^fl/fl^ and AdipoIRF4^fl/fl^ mice of both sexes (i.e., all mice). This highlighted that MDP‐mediated lowering of adipose tissue inflammation is not sufficient to improve blood glucose control. These data (and previous published data) also show that the anti‐inflammatory effect of MDP in adipose tissue is mediated by IRF4 in cells other than adipocytes (Cavallari et al., 2017). Consistent with the concept that non‐adipocyte IRF4 propagates an immune tolerizing (or anti‐inflammatory) effect in adipose tissue, a previous report showed that myeloid cell‐specific deletion of IRF4 in obese mice resulted in increased inflammatory gene expression in adipose tissue (Eguchi et al., 2013). Thus, it is likely that IRF4 in immune cells contribute to lower adipose tissue inflammation, despite loss of IRF4 in the adipocytes. A plausible model positions immune cell IRF4 as a mediator of tolerizing (i.e., anti‐inflammatory) actions in immune cells in male and female mice after treatment with MDP, whereas in adipocytes, the glucose‐lowering effects of this postbiotic require adipocyte IRF4 in male mice (Figure 4). It is not yet clear how IRF4 within the adipocyte alters blood glucose control after chronic MDP injection nor how a sex‐dependent effect alters this response in female mice. Another important limitation of this work is that we did not eliminate differences in food consumption between groups as a confounding factor mediating the glycemic response to MDP. However, consistent with all our previously published data (Cavallari et al., 2017; Cavallari et al., 2020), MDP does not alter body weight, body composition or adiposity, and it is unlikely that a reduction in food intake is an important mediator of MDP's effect on glucose homeostasis. Determining the paracrine actions of sex‐dependent factors such as estrogen and adipokines are important future goals. It was clear that MDP consistently lowered adipose tissue inflammation, but MDP did not improve blood glucose control in all mouse models (i.e., in female WT^fl/fl^ mice and male AdipoIRF4^fl/fl^ mice), which highlights that postbiotics can have effects on metabolism without necessarily altering immune responses in adipose tissue.

## AUTHOR CONTRIBUTIONS

B.M.D. and J.D.S. conceived and designed research; B.M.D, A.M.S., and D.Y.C. performed experiments; B.M.D, A.M.S., D.Y.C., and J.D.S. analyzed data; B.M.D. and J.D.S. interpreted results of experiments; B.M.D. and J.D.S. prepared figures; B.M.D. and J.D.S. drafted manuscript; B.M.D, A.M.S., D.Y.C., and J.D.S. edited, revised, and approved final version of manuscript.

## FUNDING INFORMATION

Canadian Institutes of health Research (CIHR) grant FDN‐154295.

## CONFLICT OF INTEREST

The authors have nothing to disclose.
